# Relicts of Threatened Biodiversity: Similarities and Differences among the 7230 EU Habitat Plant Communities on Montane Plateaus of Central Apennines, Italy

**DOI:** 10.3390/plants13101282

**Published:** 2024-05-07

**Authors:** Giampiero Ciaschetti, Safiya Praleskouskaya, Roberto Venanzoni

**Affiliations:** Department of Chemistry, Biology and Biotechnologies, University of Perugia, Polo Didattico V. del Giochetto 6, 06122 Perugia, Italy

**Keywords:** Apennines, phytosociology, fens, 7230 habitat

## Abstract

The habitats protected by the European Union (EU) include most peat vegetation, such as mires, swamp mires, fens, and peat bogs—all belonging to the classes *Oxycocco*–*Sphagnetea* and *Scheuchzerio*–*Caricetea fuscae* and carrying the Habitat Codes 71xx and 72xx. These types of vegetation are typical of cold and cool temperate climates, while they become rarer in Southern Europe where Mediterranean influences prevail, representing relic fragments of the past glacial climatic conditions there. Because of their limited extension and the increasing warmth and drought due to climate change, they are seriously threatened. Even if many studies were performed, their richness and distribution across Europe are still not well–understood, and only a few examples are known from the Central and Southern Apennines to date. In order to provide the syntaxonomical classification of the alkaline fens referable to the EU Habitat 7230 found on the mountain plateaus of the Central Apennines, we analyzed their species structure and flora composition, together with their chorological and ecological characteristics. We also evaluated their conservation status, pressures, and threats. The alkaline fens of the Central Apennines are found to be poorer in diagnostic species when compared to similar communities of Central and Northern Europe. However, they are rich in the species of the surrounding meadows and pastures. Among them, the new subassociation *Caricetum davallianae caricetosum hostianae* is described.

## 1. Introduction

The Habitats Directive 92/43/EEC [1] is a measure by which the European Union protects lists of natural and semi–natural habitats, wild animals, and plants, thus undertaking the goal of preserving and restoring Europe’s rich biodiversity, as outlined in the EU biodiversity strategy for 2030 [2]. Together with the Birds Directive 2009/147/EC [3], it created the Natura 2000 network, a large coordinated network of protected areas. As a directive, it required specific transposition acts by the member countries. Within the habitats listed in its Annex I, units 71xx and 72xx essentially encompass the vegetation of bogs and fens characterized by higher diversity within the classes *Oxycocco*–*Sphagnetea* Br.–Bl. et Tüxen ex Westhoff, Dijk et Passchier 1946, and *Scheuchzerio*–*Caricetea fuscae* Tx. 1937 [4,5].

In Europe, these habitats are primarily distributed in the cold and cool temperate climates, while they become rare in the southern areas where Mediterranean influences prevail. A similar difference in spreading can be observed in Italy between the northern sectors comprising the Alps and the Northern Apennines, with cool and temperate climates, and the Central and Southern regions, where the climate is warmer, with reduced summer precipitation [6].

Within the vast world of peat bogs and fens, numerous types have been distinguished, characterized by their status (active vs. dead), origin and thickness of peat (raised bogs, low, and transitional), chemistry (acidic vs. alkaline and oligotrophic/dystrophic vs. eutrophic), water supply source (ombrotrophic, soligenous, and floating bogs), and the presence or absence of woody vegetation. All these types are generally identifiable by distinctive vegetation characteristics [7]. Nevertheless, their specific composition is primarily determined by a gradient related to base saturation [8].

In this context, following the classification reported in many European syntaxonomic schemes, such as the EuroVeg checklist [8] and the Prodromus of Italian Vegetation [9], we address a broad spectrum of the alkaline fens communities, including the neutral and alkaline mountain spring fens and spring and valley mires, chiefly of the soligenic fen type [10] and characterized by high or medium base content and attributable to the order *Caricetalia davallianae* Br.–Bl. 1949 and 7230 EEC Habitat. These fens are considered very threatened in Central and Southern Italy [11], and some of them have disappeared in recent decades, as is the case regarding the Colfiorito swamp, as reported by Pedrotti [12] and verified by us. Currently, a LIFE project is underway with the ambitious goal of restoring part of the habitat there “https://www.lifeimagine.eu (accessed on 18 March 2024)”.

Even if many studies were performed on this vegetation, its richness and distribution across Europe are still not well–understood [13]. This is also true regarding Italy, where numerous plant communities belonging to the order *Caricetalia davallianae* Br.–Bl. 1949 have been reported in the Alps, the Po Valley, and the Northern Apennines, e.g., [14,15,16,17,18,19,20,21,22,23,24,25,26,27], but only a few examples [28,29,30,31,32,33,34,35,36,37,38,39] are known from the Central and Southern regions to date.

**Figure 1:**
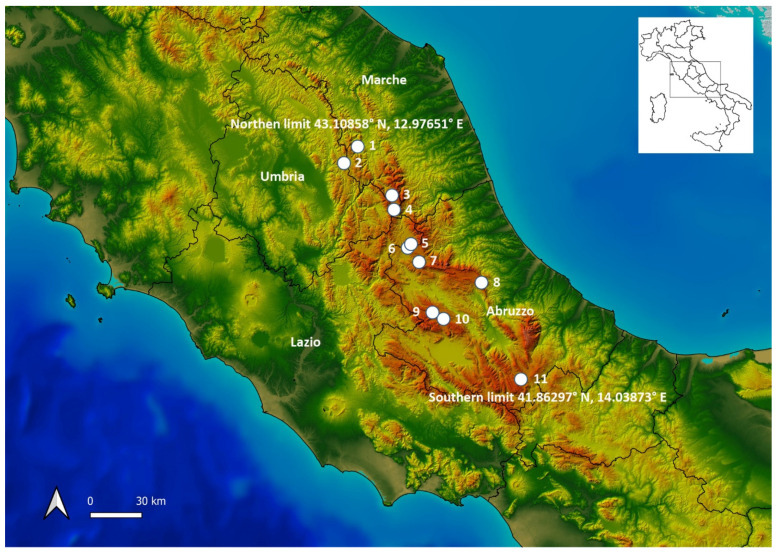
Investigated area. Sampled sites are indicated with dots: 1—Montelago, 2—Colfiorito, 3—Pian Perduto, 4—Pian Piccolo, 5 and 6—Campotosto, 7—Vomano River springs, 8—Voltigno, 9—Campo Felice, 10—Campo di Rovere, and 11—Cinque Miglia. Map source by [41].

Here, we analyze the plant communities attributable to the *Caricetalia davallianae* order as a contribution to the studies undertaken on the vegetation of the wet meadows in the montane karstic plateaus of Central Apennine, e.g., [39,40]. The investigated area and sampling sites are depicted in Figure 1. Several of these communities are reported here for the first time. This vegetation, on the plateaus of the Central Apennines, generally occupies the base of slopes where small springs occur. In these environments, also because of the high altitude, extensive grazing and hay harvesting are the only possible land use, while the agricultural exploitation is quite modest, contributing to the preservation of these fragments of vegetation.

In order to provide the syntaxonomical classification of these communities, we analyzed their floristic, biogeographic, and ecological characteristics. We also assessed their conservation status and impending and potential threats and provided considerations regarding their relict nature and vulnerability and in terms of their important role in the conservation of biodiversity.

## 2. Results and Discussion

**Figure 2:**
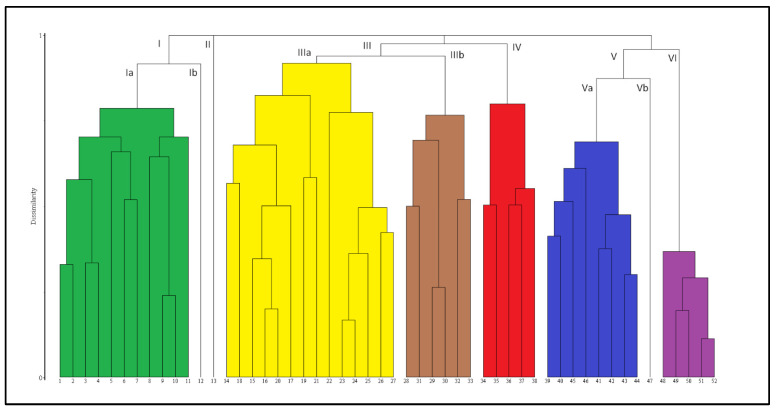
Dendrogram (similarity ratio and complete linkage) of the classification analysis of the used data.

**Figure 3:**
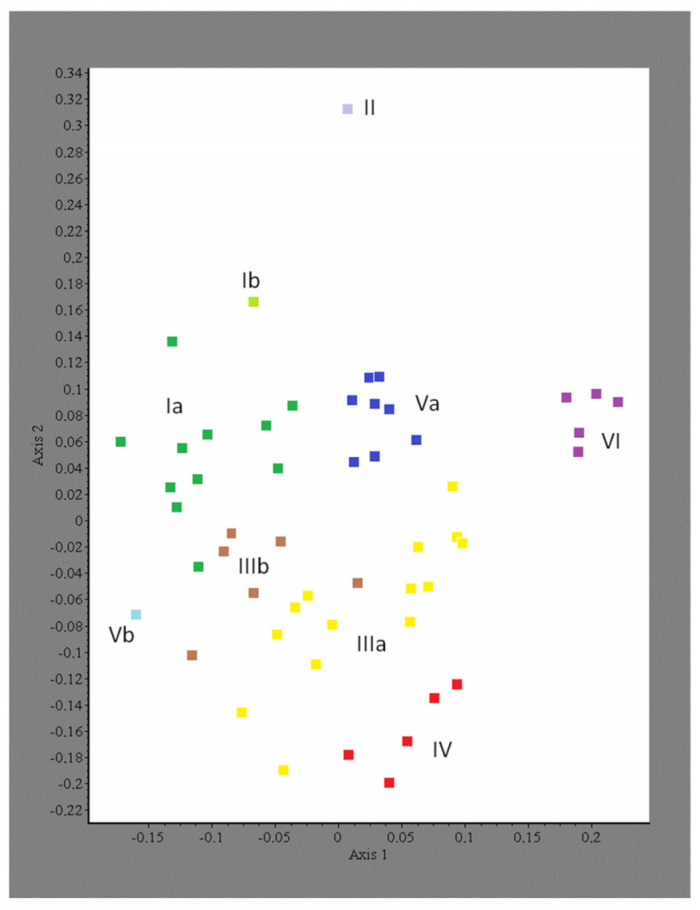
Scatterplot of phytosociological relevés according to NMDS ordination method. The Roman numerals refer to the same groups of relevés highlighted with the cluster analysis (see Figure 2).

The cluster analysis highlights six main groups of relevés (Figure 2). In Group I, one relevé distinctly deviates from the others, a separation also indicated by the NMDS analysis (Figure 3). Group II consists of a single relevé, floristically quite distinct from all the others, and also appearing isolated at a considerable distance along axis 2 in the NMDS ordination. Group III is the most comprehensive (20 relevés), with a fairly clear separation between two subgroups, IIIa and IIIb, comprising fourteen and six relevés, respectively. The NMDS ordination also shows this separation in terms of ecological gradients, although small areas of overlap appear between subgroup IIIb and subgroup IIIa and Group I.

Group IV, which is composed of five relevés, is linked to Group II; however, we prefer to treat it as a group in its own right, as also supported by the ordination (see Figure 3).

Also, Groups V and VI show a degree of similarity but at a very high fusion level in the dendrogram. Therefore, treating them as two distinct groups seems to be more appropriate, as also suggested by the separation observed in the two–dimensional ordination plot. Group V consists of nine relevés, where one of the subgroups (Vb) is markedly different from the other (Va), as suggested by both the dendrogram and the ordination plot. Group VI includes five relevés, and its identity as a group is also supported by the ordination, which places it at the right edge along axis 1.

### 2.1. Syntaxonomic Interpretation of the Groups/Clusters

#### 2.1.1. Cluster I

- *Eleocharitetum Quinqueflorae* Lüdi 1921 (Cluster Ia, Table 1, Relevés 1–11)

Structure and Floristic Composition: These are low carpets predominantly dominated by *Eleocharis quinqueflora*, characterized by the constant presence of *Blysmus compressus*, *Carex panicea*, *Juncus articulatus* subsp. *articulatus*, and, to a lesser extent, also by *C. davalliana*, *C. hostiana*, and *Potentilla erecta*. Compared to the Central European literature, the Central Apennines stands of this association exhibit a marked floristic impoverishment.

Syntaxonomy: Comparisons with associations described from Europe place the Central Apennines phytocenoses within the *Eleocharitetum quinqueflorae* Lüdi 1921 nom. mut. (Syn. *Eleocharitetum pauciflorae* Lüdi 1921), an association described in Switzerland [42] and found to be widespread from southwestern France to the Baltic Sea [43]. Some sources have listed this community from Germany, e.g., [44,45,46], Slovenia [43], Czech Republic, Slovakia, and Poland [47,48], etc. Some authors, e.g., [49,50], have used the name *Triglochino palustris–Eleocharitetum quinqueflorae* Koch (1926) 1928, which we consider to be an illegitimate synonym of the *Eleocharitetum quinqueflorae* Lüdi 1921.

Ecology: This community is often found in a mosaic with other vegetation, serving as a successional stage in water–saturated and disturbed patches of calcareous fens or areas with repetitive disturbances, such as landslide slopes or small streams, e.g., [48]. It mostly establishes near small springs, in microhabitats located on the outer portions of the plateaus, at the base of slopes, or at the contact between different lithotypes.

Chorology: In Italy, the association has been reported from some localities in the Alps and the Venetian Plain, e.g., [18,21,27]. This study represents the first record of this community for Central Italy (Figure 1: sites 1, 4, 7, 9, and 10).

EU Reference Habitat: 7230 Alkaline fens.

Conservation Status, Pressures, and Threats: The conservation status varies from bad, where only one or very few typical species were recorded, to quite good. The vegetation dominated by *Eleocharis quinqueflora* develops on peaty organic soils, with the superficial water table disturbed by natural erosion processes. If degradative processes, also due to anthropic pressure, exceed certain levels, intervention is necessary to limit them. The current state of this vegetation has been reported to the Sirente–Velino Regional Park Authority, which plans a monitoring action.

- *Eleocharitetum quinqueflorae*, Variant with *Carex oederi* and *Triglochin palustris* (Cluster Ib, Table 1, Relevé 12)

Structure and Floristic Composition: A relevé stands out from the rest of the Cluster I (Figure 2), indicating that a variant with *Triglochin palustris* and *Carex oederi* is floristically related to the *Eleocharidetum quinqueflorae*. This variant is characterized by the relatively abundant presence of *Triglochin palustris* and *Carex oederi*, along with *Plantago major* and *Agrostis stolonifera*, indicating a certain degree of anthropogenic disturbance.

Ecology: This variant develops on a mix of organic soils and rock debris with grass cover altered by livestock, agricultural machinery, and bikers. Similar aspects of the association characterized by the abundant presence of *Triglchin palustre* have been previously reported in Val d’Aosta, where it was found to be associated with cryoturbation and excessive trampling [27].

Chorology: The relevé was carried out in Campo di Rovere locality on the Altopiano delle Rocche in Abruzzo (Figure 1, site 10).

EU Reference Habitat: 7230 Alkaline fens.

Conservation Status, Pressures, and Threats: The conservation status is quite good. In addition to the threats already considered for the *Eleocharidetum quinqueflorae*, this variant is subject to pressure from the trampling by cattle, horses, and agricultural vehicles, which may threaten the presence of the habitat if not limited.

#### 2.1.2. Cluster II

- *Carex canescens* subsp. *canescens community* (Cluster II, Table 2)

Structure and Floristic Composition: *Carex canescens* L. subsp. *canescens* is a taxon of a temperate cosmopolitan distribution, common in the Alps but very rare in peninsular Italy [51]. In the surveyed area, it forms nearly continuous swards that are extremely poor in floristic terms: only three other vascular species, *Glyceria notata*, *Persicaria amphibia,* and *Nardus stricta*, were recorded in this community.

Syntaxonomy: *Carex canescens* subsp. *canescens* is considered a diagnostic species of the *Caricion fuscae* alliance (*Caricetalia fuscae*), and it was used as an eponymous taxon in the *Caricion canescenti*–*nigrae* Nordhagen ex Tx. 1937 corr. Timmermann in Dengler et al. 2004. The species also provides its name to the alliance *Sphagno*–*Caricion canescentis* Passarge (1964) 1978 nom. conserv. propos, related to ‘poor fens’ and to the association *Carici*–*canescentis*–*Agrostietum caninae* Tx. 1937, now included in the *Caricetum nigrae* Braun 1915 [48]. In Italy, *Carex canescens* dominates the communities of the Tuscan–Emilian Apennines, attributed to these latter associations [52,53].

Ecology: The relevé was recorded in the central part of a small water body receiving water from neighboring meadows, where *Carex canescens* forms a sward, likely interpretable as a relictual testimony of the past presence of a bog. The abundance of *Carex canescens* indicates the only possibility for this species to survive in this small wetland area. The extreme floristic poverty, together with the presence of *Nardus stricta*, abundant in the surrounding meadows, suggest a certain level of soil acidity and organic matter accumulation due to grazing.

Chorology: The only relevé was carried out on the Voltigno Plateau, located on the Gran Sasso Massif (Figure 1, site 8). Although the presence of the species is also documented in Calabria [54], this represents the southernmost *Carex canescens* subsp. *canescens* vegetation known in Italy.

EU Reference Habitat: The correspondence to an EU 43/92 Directive habitat is challenging. It is a low–grown, sunny, acidophilic bog, similar to a floating mat due to the presence of ground water, lacking a specific code among EU habitats. Generally, referencing to Habitat 7230 is hindered by the absence of the base–loving species of the *Caricion davallianae*. Similarly, a reference to Habitat 7140, although indicated in the Natura 2000 form, is unlikely due to the absence of sphagnum mosses.

Conservation Status, Pressures, and Threats: The trampling and excretions of domestic livestock, especially cattle, constitute strong negative edaphic and physical pressure on this rare and already impoverished plant community, threatening its survival. Therefore, it would be advisable to protect it with a specific fence.

#### 2.1.3. Cluster III

- *Caricetum davallianae* Dutoit 1924 (Cluster IIIa, Table 3, rell. 1–13):

Structure and floristic Composition: The vegetation stands attributed to this association are small in size, as indicated by the term “parvocaricetum” used in the past by some authors indicating communities dominated by small sedges like this. *Carex davalliana* is always more or less dominant, along with *Carex panicea* and sometimes *Eriophorum latifolium* or *Carex echinata* subsp. *echinata*. Other species typical of low alkaline bogs are present but with rather low frequencies. In addition to *C. davalliana*, the only taxa recorded in at least half of the relevés are *Potentilla erecta*, *Blysmus compressus*, and *Juncus articulatus* subsp. *articulatus*. Other common taxa include *Ranunculus acris*, *Trifolium pratense*, *Briza media*, *Juncus inflexus* subsp. *inflexus*, and *Equisetum palustre*.

Syntaxonomy: The scarcity of stands, combined with general floristic impoverishment, make it challenging to clearly classify them into one of the plant associations described in the literature, e.g., [55,56,57]. This problem is likely correlated with the geographical context due to its occurrence at the limit or outside the range of several diagnostic species. A similar impoverishment often occurs in other territorial contexts when the association descends from its typical elevational range [58]. For these reasons, we choose to classify the relevés from Central Apennines in the *Caricetum davallianae* Dutoit 1924 association.

Relevé n. 14 of Table 3 was already reported as a community of *Carex echinata* [35]. Based on the cluster analysis, it is to be included in the *Caricetum davallianae*. However, since *Carex davalliana* is absent, it can be considered as an impoverished variant of this association.

Ecology: These communities develop at the edges of small water flows near small springs, mostly on the marginal position portions of the plateaus.

Chorology: The *Caricetum davallianae* is widespread in Europe [59], and it was already recorded in Italy in the Alps, e.g., [18,21,24,60]. The relevés in Table 3 come from Pian Perduto, Pian Piccolo of Castelluccio, the surroundings of Campotosto Lake, and the nearby springs of the Vomano River between Gran Sasso and Monti della Laga (Figure 1, sites 3, 4, 5–6, and 7).

EU Reference Habitat: 7230 Alkaline fens.

Conservation Status, Pressures, and Threats: The conservation status, in all the observed localities, appears to be unfavorable. These phytocenoses have a relictual character and, consequently, cover very limited surfaces. The main threats come from the potential tapping of spring waters and grazing, especially with large animals (e.g., cattle or horses).

- *Caricetum davallianae* Dutoit 1924 c*aricetosum hostianae subass. nova hoc loco* (Cluster IIIb, Table 3, rel. 15–20, holotype rel. 16 of Table 3)

Structure and Floristic Composition: Within the *Caricetum davallianae* Dutoit 1924, relevé group 15–20 is characterized by the abundant presence of *Carex hostiana* DC. This species is rare in Central Italy and is limited to a few stands in the Central Apennines, representing the southern distribution limit of this species in Italy [61,62]. In addition to *Carex hostiana*, *Dactylorhiza incarnata* subsp. *incarnata* and *Eleocharis quinqueflora* differentiate this relevé group from the previous one. The other well–represented taxa are *Carex panicea*, *Potentilla erecta*, *Blysmus compressus*, *Juncus articulatus* subsp. *articulatus*, and, among those typical of other phytosociological classes, also *Juncus inflexus* subsp. *inflexus*, *Equisetum palustre*, *Succisa pratensis*, and *Carex flacca* subsp. *flacca*.

Syntaxonomy: *Carex hostiana* is often present in communities of the *Caricion davallianae* Klika 1934 alliance and was indicated as “more or less characteristic” in the original diagnosis of *Caricetum davallianae* Dutoit 1924 [63]). Nevertheless, several authors, such as Issler [64], Hallberg [65]), Rodwell [66], and Trinajstić [67], proposed associations in those situations where the species realizes high cover values. Regarding our relevés, we believe that they cannot be attributed to any of these associations and therefore propose a new subassociation to be described within *Caricetum davallianae* Dutoit 1924.

**Figure 4:**
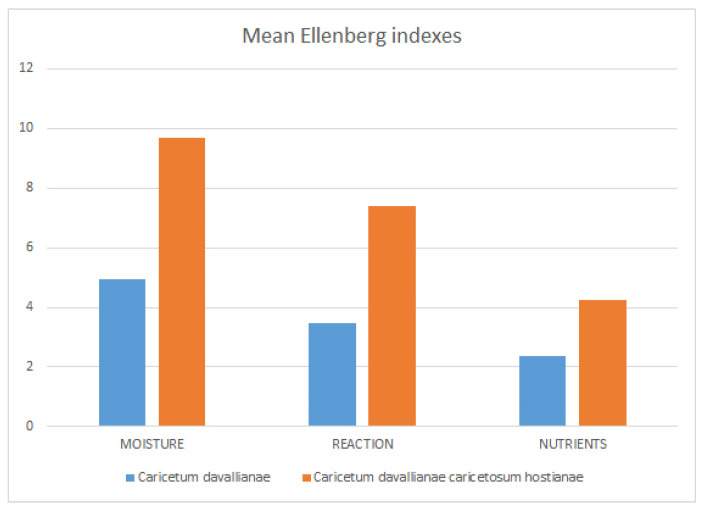
*Caricetum davallianae* and *C. davallianae caricetosum hostianae* compared by means of Ellenberg’s indexes of moisture, reaction, and soil nutrients.

Ecology: The stands of subassociation *caricetosum hostianae* are found near small slope springs, in flat or sub–flat situations. In the context of this research, it proves to thrive under the conditions of higher humidity, lower acidity, and higher nitrogen content when compared to the typical association, as highlighted by the analysis using Ellenberg indicator values [68], calculated based on species abundance (Figure 4).

Chorology: The relevés attributed to *Caricetum davallianae caricetosum hostianae* all come from Campo di Rovere, in the territory of Rocca di Mezzo, and from the border area between Poggio Cancelli (Campotosto) and Aringo (Montereale), all in the province of L’Aquila (Abruzzo Region) (Figure 1, sites 6 and 10).

EU Reference Habitat: 7230 Lowland alkaline fens.

Conservation Status, Pressures, and Threats: These communities are quite well–preserved. At Campo di Rovere, the area occupied by Habitat 7230, which is the mosaic composed by these communities together with others referable to the same habitat, is greater than in the other investigated sites. However, it is not exempt from threats and should soon be protected by the Regional Park Sirente–Velino, which plans a monitoring action. The disturbance pressures and threats are linked to water withdrawal, as well as overgrazing and the abandonment of haymaking.

#### 2.1.4. Cluster IV

- *Eriophorum latifolium community* (Cluster IV, Table 4)

**Structure and Floristic Composition:** These are meadows with continuous cover dominated by *Eriophorum latifolium*, frequently accompanied by *Carex panicea*, *Ranunculus repens*, *Juncus inflexus* subsp. *inflexus*, and *Briza media*. At the upper plateau of Montelago (Marche Region), *Carex lepidocarpa* subsp. *lepidocarpa*, *Carex distans*, and *Carex flacca* subsp. *flacca* are also common. In Table 4, a floristic impoverishment in the alliance, order, and class diagnostic species can be observed, being even greater than in the other discussed vegetational types.

**Syntaxonomy:** *Eriophorum latifolium* is considered to be a diagnostic species of the *Caricion davallianae* alliance, e.g., [9,48]. In Italy, as in the rest of Europe, this showy species is documented both in the vegetation of this alliance, e.g., [37,69,70,71,72], and in the communities referring to other syntaxonomic units of the class *Scheuchzerio*–*Caricetea fuscae*, e.g., [13,15,26,48,70,73]. It was found in the meadows of the *Molinio*–*Arrhenatheretea* class too, e.g., [29,73,74], and in wetland communities, e.g., [15]. Several associations with *Eriophorum latifolium* within the *Caricetalia davallianae* order were reported from Europe, e.g., [70,71,75,76,77], to which our relevés do not seem to be referable. Phytocenoses with a dominant or co–dominant *Eriophorum latifolium* have also been found in various locations in Italy and have been referred to several syntaxonomic units. Pedrotti [29] reported the *Eriophoretum latifolii* from Piani di Montelago, considering this a provisional name and citing only some species in the floristic composition. Even if Pedrotti and Pettorossi [31] used the same association name at the Palude di Colfiorito (without relevés), Pedrotti and Sanesi [30] confirmed in the same volume that it is a provisional name. Moreover, recently, Pedrotti [12], based on an old relevé, referred only to a generic community with *Eriophorum latifolium* for the same location. The name *Eriophoretum latifolii* Pedrotti 1969 is therefore invalid based on art. 3b of the ICNP [78]. Consequently, the use of that name by Gerdol and Tomaselli [17] in the Apuan Alps has to be considered incorrect, and their proposal regarding the subassociation name *Eriophoretum latifolii* Pedrotti 1969 *cratoneuretosum commutati* Gerdol et Tomaselli 1987 has to be considered invalid too (art. 4a ICPN). Mariotti [79]) reported the presence of the *Carici paniculatae*–*Eriophoretum latifoliae* O. Bolòs et Vives in O. Bolòs 1956 in Liguria, classified into the *Magnocaricion elatae* Koch 1926. Raffaelli et al. [53] and Foggi et al. [26] mentioned a community of *Eriophorum latifolium* in the Tuscan–Emilian Apennines, both classifying them in the *Caricion nigrae* (acidophilous fens). Another *E. latifolium* community was reported from the plateau of Folgaria (Trentino) [60], without clear syntaxonomic placement. Pirone [37] described, for the northern slope of the Gran Sasso Massif, subassociation *eriophoretosum latifolii* of the *Pinguiculo vulgaris*–*Caricetum praetutiane*, an association proposed by Biondi et al. [36] for the same Massif.

The poverty in the diagnostic species of alliance, order, and class, as highlighted, does not allow us to attribute our relevés to a specific association but only to propose the recognition of a generic community within the *Caricion davallianae*.

**Ecology:** These are peaty meadows mostly located at the base of spring slopes, along and around narrow water streams, with more or less continuous surface water flow.

**Chorology:** The community of *Eriophorum latifolium* was found at the sources of the Vomano River (Abruzzo) and upper plateau of Montelago (Marche) (Figure 1, sites 1 and 7), while the one reported at Colfiorito swamp [12,30,31] (Figure 1, site 2) has to be considered extinct.

**EU Reference Habitat:** 7230 Alkaline lowland fens.

**Conservation Status, Pressures, and Threats:** The conservation status of these phytocenoses is considered unfavorable due to the small areas occupied and the floristic poverty in the diagnostic species. Among the pressures and threats, grazing, especially by cows, and possible variations in the soil water regimes, are to be considered.

#### 2.1.5. Cluster V

- *Blysmus compressus community* (Cluster Va, Table 5)

Structure and Floristic Composition: These are small–sized meadows whose floristic composition does not differ very clearly from that of the other communities already described. However, they differ from these, like the communities attributed to the *Eriophorum latifolium* community, by their floristic poverty. In fact, many of the diagnostic species of alliance and class (e.g., *Carex davalliana*, *Eleocharis quinqueflora*, *Carex hostiana,* and *Potentilla erecta*), rather present in the other vegetation types, are almost absent here, probably due to the state of degradation caused by grazing.

Syntaxonomy: Few communities of the *Caricetalia davallianae* with dominant or co–dominant *Blysmus compressus* are known in the literature, e.g., [80,81,82,83], mostly referring to the *Carici*–*Blysmetum compressi* Eggler 1933, described from Austria [84] and also reported from other countries such as Germany [85], Bosnia [86], Croatia [87], Slovenia [88], and Ukraine [83]. Hájek et al. [89] noted, however, that the name of the association is invalid and that the name *Carici flavae*–*Blysmetum compressi* Coldea 1977 is unapplicable. Therefore, they classified the Carpathian relevés that had been referred to this association partly to the *Carici flavae*–*Cratoneuretum filicini* Kovács et Felföldy 1960 and partly to the *Valeriano simplicifoliae*–*Caricetum flavae* Pawłowski et al. 1960. We do not believe that our relevés can be attributed to any of these associations, and even a possible proposal for a new association is poorly supported by the data. Communities dominated by *Blysmus compressus*, and attributed to the *Caricion fuscae*, have also been found in Italy, in Val d’Aosta [27].

Ecology: This vegetation is established in small water spring habitats or along watershed lines, often in a mosaic with other fen or meadow communities, in more or less heavily grazed sites. Grazing causes both a heavy disturbance due to trampling and soil enrichment in the organic matter, as evidenced by the abundance of nutrient–demanding species such as *Carex hirta* and *Ranunculus repens*. These communities, in particular the most impoverished aspects, are transitional forms towards the hygro–nitrophilous communities of the *Potentillion anserinae*.

Chorology: These communities were found at Campo Felice, in the Sirente–Velino mountain group, at Voltigno on the Gran Sasso, and between Campotosto and Amatrice on the Monti della Laga (Figure 1, sites 5, 8, and 9.)

EU Reference Habitat: 7230 Alkaline fens.

Conservation Status, Pressures, and Threats: The conservation status is unfavorable as these communities are already very impoverished compared to their potential. The limited extension, often just above the sum of the surveyed areas, makes these phytocenoses particularly vulnerable. Bovine and equine grazing, sometimes intense, is the main contingent pressure, while, among the medium to long–term threats, a possible decrease in water availability plays a significant role.

- *Menyanthetum trifoliati* Steffen 1931 (Cluster Vb, Table 6)

Structure and Floristic Composition: This is a wetland community where *Menyanthes trifoliata* is clearly dominant. The floristic composition includes various taxa of the *Caricion/Caricetalia davallianae*, such as *Blysmus compressus*, *Juncus articulatus* subsp. *articulatus*, *Triglochin palustris,* and *Dactylorhiza incarnata* subsp. *incarnata*. Relatively abundant are also *Equisetum palustre*, *Mentha aquatica* subsp. *aquatica*, *Caltha palustris,* and *Juncus inflexus* subsp. *inflexus*.

Syntaxonomy: *Menyanthes trifoliata* L. is a rare species in Italy, especially along the peninsula, where it is very localized [90]. This plant is often present, even as a dominant species, in communities classified within the *Phragmito*–*Magnocaricetea*, the *Littorelletea uniflorae*, and the *Scheuchzerio*–*Caricetea*, e.g., [48,72,88,91,92]. Within the latter class, it is indicated by various authors as a characteristic of various syntaxa, e.g., [48,93,94,95]. Also in Italy, *Menyanthes trifoliata* communities, reported from the Dolomites [18], Calabria [38], and the Northern Apennines [26,52,53,69], have been referred to different syntaxa classified into different classes.

Comparing our relevé with the different associations described, it seems appropriate to refer this unit to the *Caricion davallianae* Klika 1934, and namely to the *Menyanthetum trifoliatae* Steffen 1931. Some affinity was also found with the *Eriophoro latifolii*–*Menyanthetum trifoliati* Redžić in Redžić, Trakić et Barudanović 2013, where, however, *Eriophorum latifolium* is missing.

Ecology: This community was found on flat terrain, with 5–10 cm of surface water in midsummer.

Chorology: The only relevé attributed to this vegetation was recorded at the sources of the Vomano River in Abruzzo (Figure 1, site 7).

EU Reference Habitat: 7230 Alkaline fens.

Conservation Status, Pressures, and Threats: The vegetation with *Menyanthes trifoliata* can currently be considered to have a decent conservation status, especially because the occupied area is relatively widespread, at least compared to the other communities treated here. Early mowing of meadows and grazing are the major current pressures. The location in a flat area very close to a major communication route such as the S.S. 80 makes this community threatened by possible infrastructural expansions. In the past, tourist buildings had already been built a few tens of meters away, along the road.

#### 2.1.6. Cluster VI

- *Carex nigra* subsp. *nigra community* (Cluster VI, Table 7)

Structure and Floristic Composition: The relevés documenting this relictual vegetation were already published by Ciaschetti et al. [39], to which we refer for the description.

Syntaxonomy: The only presence of *Carex nigra* subsp. *nigra* and *C. panicea* among the diagnostic taxa of the *Scheuchzerio*–*Caricetea* does not allow a clear classification of this community. Based on the absence of clearly basophilus species and the presence of acidophilous elements as *Carex nigra* subsp. *nigra*, *Ranunculus pollinensis*, and *Danthonia decumbens* subsp. *decumbens*, we prefer to classify this community in the *Caricion fuscae/Caricetalia fuscae* rather than in the *Caricetalia davallianae* as in [39].

Ecology: This community is established on peaty and acidic soils, wet for almost the whole year, in the lowest portion of a large mountain plateau.

Chorology: The relevés of Table 7 were carried out on the Cinque Miglia Plateau in Abruzzo (Figure 1, site 11).

EU Reference Habitat: Such a sunny mire, showing an acidophilic character, is not included in the habitats of Directive 92/43/EEC.

Conservation Status, Pressures, and Threats: This community has rather small stands, reaching only a few tens of square meters. It is currently mowed once a year, in the middle of summer, which reduces the possibility of the dispersal of many plant species. In the immediate vicinity, in addition to hay meadows, there are crops including mainly potatoes, cereals, and legumes for grain, whose cultivation can cause disturbance. As for the other plant communities reported here, changes in soil water regime constitute the main medium– to long–term threat.

### 2.2. Syntaxonomic Scheme

*SCHEUCHZERIO PALUSTRIS*–*CARICETEA FUSCAE* Tx. 1937

# *Caricetalia davallianae* Br.–Bl. 1949.

*§ Caricion davallianae* Klika 1934

*Eleocharitetum quinqueflorae* Lüdi 1921

*Carex oederi* and *Triglochin palustris* variant

*Caricetum davallianae* Dutoit 1924

*Caricetum davallianae* Dutoit 1924 *caricetosum hostianae* Ciaschetti, Praleskouskaya, R. Venanzoni 2024

*Menyanthetum trifoliatae* Steffen 1931

*Eriophorum latifolium* community

Community of *Blysmus compressus* community

# *Caricetalia fuscae* Koch 1926

*§ Caricion fuscae* Koch 1926 nom. conserv. propos.

*Carex canescens* community

*Carex nigra* subsp. *nigra* community

## 3. Materials and Methods

The data primarily consist of unpublished relevés, supplemented by other published sources related to similar communities found in the biogeographic context of the Central Apennines and particularly limited to the high–elevation plateaus. For a detailed list of the data sources, including the dates and localities of the relevés, see Appendix B. Pictures of the surveyed plant communities are shown in Supplementary Files Figures S1–S6.

Both the original and published relevés were carried out using the classical field sampling method of the Zürich–Montpellier school [96]. The abundance–dominance values, assigned according to the original Braun–Blanquet’s scale, were converted into the ordinal one according to Van der Maarel [97]. The resulting matrix (132 species × 52 samples) was processed using the multivariate analysis package MatEdit [98], downloadable at “www.vegitaly.it (accessed on 19 December 2023)”, with the similarity ratio on cover values as the resemblance index. A cluster analysis was performed using complete linkage. An NMDS ordination was also conducted with the SYNTAX 2000 package [99] using the chord distance as the dissimilarity coefficient. The names of the species follow the checklist of the Italian Flora as listed in the Portal to the Flora of Italy [54], while the reference for the syntaxonomical scheme is the Vegetation Prodrome of Italy [9].

## 4. Conclusions

The vegetation of the alkaline fens mostly classified in the order *Caricetalia davallianae* Br.–Bl. 1950 is present in the Central Apennines despite its landscape–dominating calcareous matrix, where it takes refuge in the extensive system of tectonic–karstic plateaus. We suggest that it used to be much more widespread during the Quaternary glaciations in the Apennines; today, this vegetation is confined to very few environments that remain suitable [100], where it persists in the form of extremely small stands, often at the limit of monitoring possibility, in highly simplified vegetational patterns, and mostly with a characteristic floristic composition reduced to a few species [101].

On the plateaus of the Central Apennines, as mentioned above, this vegetation occupies the base of the slopes near small springs or along water catchment lines. In some cases, this vegetation is present in the center of the plateau, near small bodies of water, or along the demarcation line between two different lithotypes that generate a rise in the water table. The position in the geo–morphological profile, together with the elevation (montane belt), likely contributed to the preservation of these fragments of vegetation as extensive grazing and hay harvesting have been the only possible land use in these environments while agricultural exploitation has been quite a modest threat.

Compared to similar communities in Central and Northern Europe, the Central Apennine populations show an impoverished floristic composition, highlighted both by the low number of diagnostic species and by the conspicuous presence of species from the surrounding meadows and pastures. This, along with the very small size of the occupied surfaces, make these communities extremely vulnerable to disturbance pressures and threats. These are mainly posed by intense grazing, especially by large animals, and variations in the water regime that could be due to both water withdrawals and a general decrease in precipitation, especially in the summer. Unlike other habitats that are more widespread and better–structured, the EU “7230 Lowland alkaline fens” habitat is therefore at a high risk of disappearing in the Apennine biogeographical context, as already occurred regarding the Colfiorito swamp [12], and deserves specific conservation actions.

## Figures and Tables

**Table 1:** *Eleocharidetum quinqueflorae* Lüdi 1921.

Relevé No.	1	2	3	4	5	6	7	8	9	10	11	12
Cover (%)	80	80	85	85	70	85	90	90	95	90	100	100
Sampled area (in sq.m)	4	1	4	4	4	4	4	1	6	10	1	4
Elevation (in m above sea level)	1350	1350	1300	1300	1170	1300	1300	1350	1530	1530	923	1300
*Eleocharitetum quinqueflorae* and upper units												
*Eleocharis quinqueflora*	2	3	5	5	3	4	5	4	5	5	4	1
*Blysmus compressus*	4	2	2	2	.	1	+	.	1	+	.	.
*Carex panicea*	1	1	.	.	1	1	1	.	1	2	1	.
*Juncus articulatus* subsp. *articulatus*	1	1	.	1	1	1	1	.	1	2	.	+
*Potentilla erecta*	+	+	1	1	1	.	.	.	.	.	.	.
*Carex davalliana*	+	.	1	1	2	.	.	.	.	.	.	.
*Carex hostiana*	.	.	2	2	+	+	2	.	.	.	.	.
*Epilobium palustre*	.	.	+	+	.	.	.	.	.	.	.	.
*Parnassia palustris* subsp. *palustris*	.	.	1	.	1	.	.	.	.	.	.	.
*Dactylorhiza incarnata* subsp. *incarnata*	.	.	.	.	+	+	.	.	.	.	.	.
*Taraxacum sect. Palustria*	.	.	.	.	.	.	.	.	+	.	2	.
*Carex oederi* and *Triglochin palustris* variant												
*Carex oederi*	.	.	.	1	.	2	.	.	.	.	.	2
*Triglochin palustris*	.	.	.	.	.	.	1	.	.	.	.	2
Other taxa												
*Carex flacca* subsp. *flacca*	1	+	+	+	.	.	.	.	1	+	1	1
*Trifolium repens*	+	.	1	.	.	1	+	2	.	.	.	+
*Ranunculus repens*	1	.	.	.	.	.	.	.	2	1	.	2
*Juncus inflexus* subsp. *inflexus*	.	.	+	+	.	+	+	.	.	.	.	+
*Rhinanthus minor*	.	.	+	.	+	+	+	.	.	.	.	.
*Scorzoneroides autumnalis*	.	.	.	.	+	.	.	1	2	1	.	.
*Glyceria notata*	1	+	1	.	.	.	.	.	.	.	.	.
*Hypericum tetrapterum*	1	.	.	.	+	+	.	.	.	.	.	.
*Lotus pedunculatus*	.	.	+	.	.	+	1	.	.	.	.	+
*Molinia caerulea*	.	.	.	+	1	+	.	.	.	.	.	.
*Carex hirta*	.	.	.	.	.	.	1	.	1	+	.	.
*Deschampsia caespitosa* subsp. *caespitosa*	.	.	.	.	.	.	.	1	1	1	.	.
*Ranunculus acris* s.l.	+	.	.	.	.	.	2	.	.	.	.	.
*Bellis perennis*	1	.	.	.	.	.	.	1	.	.	.	.
*Succisa pratensis*	.	.	1	1	.	.	.	.	.	.	.	.
*Briza media*	.	.	.	+	.	.	.	.	.	.	+	.
*Equisetum palustre*	.	.	.	.	+	1	.	.	.	.	.	.
*Agrostis castellana*	.	.	.	.	.	.	.	.	2	2	.	.
Species of low frequency	0	0	5	0	3	3	3	3	1	2	5	5

**Table 2:** *Carex canescens* subsp. *canescens* community.

Cover (%)	100
Sampled area (in sq.m)	16
Elevation (in m above sea level)	1360
*Carex canescens* subsp. *canescens*	5
*Glyceria notata*	2
*Nardus stricta*	1
*Persicaria amphibia*	2

**Table 3:** *Caricetum davallianae* Dutoit 1924.

Releve No.	1	2	3	4	5	6	7	8	9	10	11	12	13	14	15	16*	17	18	19	20
Cover (%)	90	90	100	100	100	100	100	90	100	100	100	100	100	100	100	100	100	100	100	100
Sampled area (in sq.m)	16	16	16	8	10	9	16	9	10	10	10	10	10	9	4	4	16	4	16	16
Elevation (in m above sea level)	1260	1340	1340	1340	1452	1140	1350	1140	1452	1452	1452	1452	1452	1330	1300	1300	1300	1300	1330	1330
*Caricetum davallianae*																				
*Carex davalliana*	4	4	4	3	4	4	4	2	+	+	2	2	3		4		2		+	2
variant of Carex echinata subsp. *echinata*																				
*Carex echinata* subsp. *echinata*	1	.	1	.	.	.	.	.	.	.	+	.	.	2	.	.	.	.	.	.
subassociation *caricetosum hostiane*																				
*Carex hostiana*	.	.	.	.	.	2		+							1	4	4	4	1	1
*Dactylorhiza incarnata* subsp. *incarnata*						1									+	+	+		+	1
*Eleocharis quinqueflora*			+												1	+		2	1	
Upper units																				
*Carex panicea*	2	2	2	2	2	+	1	1	3	3	3	1	1	1	.	2	+	3	1	2
*Potentilla erecta*	.	1	2	1	.	1	3	1	.	.	.	+	+	2	2	1	2	2	+	.
*Blysmus compressus*	1	2	1	1	.	.	1	.	.	.	.	+	.	+	.	2	2	2	1	.
*Juncus articulatus* subsp. *articulatus*	+	1	1	+	.		2							1			+	1	3	2
*Eriophorum latifolium*				3		+		3											+	
*Taraxacum* Sect. *Palustria*					+				+		+	+								
*Epipactis palustris*						1		1												
*Parnassia palustris* subsp. *palustris*								1										+		
*Carex nigra* subsp. *nigra*																			3	3
*Carex oederi*		+																		
*Menyanthes trifoliata*						1														
Other species																				
*Ranunculus acris* s.l.	+	1	.	+	+	.	+	.	+	.	+	1	+	.	+	.	1	+	.	+
*Trifolium pratense* s.l.	.	+	+	+	+	.	+	.	.	.	+	+	.	.	+	.	1	.	+	1
*Briza media*	+	2	2	1	.	.	1	.	.	.	+	.	+	.	.	+	1	.	.	1
*Juncus inflexus* subsp. *inflexus*	+	+	.	.	.	+	.	+	.	.	.	.	.	.	1	+	+	1	3	2
*Equisetum palustre*	1	.	.	2	+	2	.	.	.	.	+	.	+	.	.	+	.	1	1	2
*Ranunculus repens*	+	.	+	.	.	.	.	.	2	2	+	1	1	+	.	.	.	.	.	1
*Deschampsia caespitosa* subsp. *caespitosa*	.	.	.	.	1	.	.	.	+	1	1	1	1	1	.	.	+	.	.	+
*Carex acuta*	.	1	.	1	.	.	.	.	1	1	+	+	+	+	.	.	.	.	.	.
*Scorzoneroides autumnalis*	.	.	.	.	1	+	.	.	2	2	2	1	2	+	.	.	.	.	.	.
*Succisa pratensis*	.	.	.	.	.	2	.	1	.	.	.	.	.	.	1	1	2	+	+	+
*Carex flacca* subsp. *flacca*	.	.	+	.	.	.	1	.	.	.	.	.	.	.	1	1	1	1	.	1
*Trifolium repens*	.	.	.	.	.	.	.	.	+	+	.	.	+	.	.	+	1	.	1	+
*Ranunculus auricomus* agg.	+	.	.	.	.	.	.	.	1	1	1	+	1	.	.	.	.	.	.	.
*Ranunculus flammula*	.	.	1	.	.	.	+	.	+	+	.	.	+	+	.	.	.	.	.	.
*Rhinanthus minor*	.	.	.	.	.	.	.	.	.	+	.	.	+	.	1	+	+	+	.	.
*Hypericum tetrapterum*	.	1	+	+	.	.	1	.	.	.	.	.	.	.	.	.	.	.	.	1
*Linum catharticum* s.l.	.	.	+	.	+	.	.	.	.	.	.	.	+	.	.	.	.	+	.	+
*Molinia caerulea*	.	.	.	.	.	1	.	1	.	.	.	.	.	.	+	+	.	+	.	.
*Mentha aquatica* subsp. *aquatica*	1	.	.	.	.	.	.	.	.	.	.	.	.	.	+	.	+	.	1	.
*Bellis perennis*	.	1	.	+	+	.	.	.	.	.	.	.	.	.	.	.	.	.	+	.
*Dactylorhiza maculata* subsp.*saccifera*	.	.	.	.	.	.	.	+	.	.	.	.	.	.	1	.	+	.	.	+
*Galium debile*	.	.	.	.	.	.	.	.	+	+	+	+	.	.	.	.	.	.	.	.
*Scorzoneroides cichoracea*	.	.	.	.	.	.	.	.	1	1	.	1	+	.	.	.	.	.	.	.
*Lathyrus pratensis* subsp. *pratensis*	+	.	.	.	+	.	.	.	.	.	.	.	.	.	.	.	.	.	.	1
*Glyceria notata*	.	1	.	.	.	.	.	.	.	.	.	.	.	.	.	.	+	.	.	1
*Festuca rubra* s.l.	.	.	.	.	1	.	.	.	.	.	.	.	+	.	.	.	+	.	.	.
*Eleocharis palustris* subsp. *palustris*	.	.	.	.	.	.	.	.	+	.	+	1	.	.	.	.	.	.	.	.
*Trifolium badium*	.	.	.	.	.	.	.	.	.	1	+	.	+	.	.	.	.	.	.	.
*Lotus pedunculatus*	.	.	.	.	.	.	.	.	.	.	.	.	.	.	+	+	.	+	.	.
*Mentha arvensis*	.	.	.	.	.	.	.	.	.	.	.	.	.	.	.	+	+	+	.	.
*Holcus lanatus* subsp. *lanatus*	+	.	.	.	.	.	.	.	.	.	.	.	.	.	.	.	.	.	.	1
*Galium verum* subsp. *verum*	.	.	.	.	+	.	.	.	.	.	+	.	.	.	.	.	.	.	.	.
*Ranunculus montanus* agg.	.	.	.	.	+	.	.	.	.	.	.	+	.	.	.	.	.	.	.	.
*Gymnadenia conopsea*	.	.	.	.	.	+	.	+	.	.	.	.	.	.	.	.	.	.	.	.
*Vicia cracca*	.	.	.	.	.	+	.	.	.	.	.	.	.	.	.	.	+	.	.	.
*Galium palustre* subsp. *palustre*	.	.	.	.	.	.	.	.	.	.	.	.	.	+	.	.	+	.	.	.
*Alchemilla* sp.	.	.	.	.	.	.	.	.	.	.	.	.	.	.	+	+	.	.	.	.
*Euphrasia salisburgensis*	.	.	.	.	.	.	.	.	.	.	.	.	.	.	+	+	.	.	.	.
*Gentiana pneumonanthe* subsp. *pneumonanthe*	.	.	.	.	.	.	.	.	.	.	.	.	.	.	+	.	+	.	.	.
*Phleum nodosum*	.	.	.	.	.	.	.	.	.	.	.	.	.	.	.	.	+	.	.	1
Species of low frequency	3	0	0	0	2	1	0	2	1	0	0	0	1	5	1	1	1	1	3	6

**Table 4:** *Eriophorum latifolium* community.

Releve No.	1	2	3	4	5
Cover (%)	90	100	97	99	100
Sampled area (in sq.m)	4	4	4	4	4
Elevation (in m above sea level)	1140	1140	924	922	923
*Eriophorum latifolium* community and upper untis					
*Eriophorum latifolium*	3	4	4	3	3
*Carex panicea*	.	1	2	1	2
*Carex lepidocarpa* subsp. *lepidocarpa*	.	.	1	1	1
*Epipactis palustris*	1	2	.	.	.
*Dactylorhiza incarnata* subsp. *incarnata*	1	.	.	.	.
*Potentilla erecta*	+	.	.	.	.
*Parnassia palustris* subsp. *palustris*	.	+	.	.	.
*Carex oederi*	.	.	2	.	.
*Eleocharis quinqueflora*	.	.	.	+	.
Other species					
Ranunculus repens	2	+	+	+	1
*Juncus inflexus* subsp. *inflexus*	1	+	+	2	1
*Briza media*	2	2	.	1	1
*Lathyrus pratensis* subsp. *pratensis*	+	.	.	+	+
*Carex distans*	.	.	1	1	1
*Carex flacca* subsp. *flacca*	.	.	3	1	1
*Carex paniculata* subsp. *paniculata*	2	2	.	.	.
*Hypericum tetrapterum*	1	1	.	.	.
*Vicia cracca*	+	1	.	.	.
*Pulicaria dysentherica*	+	1	.	.	.
*Prunella vulgaris* subsp. *vulgaris*	+	+	.	.	.
*Equisetum palustre*	.	3	+	.	.
*Centaurea jacea* s.l.	.	.	+	2	.
*Filipendula ulmaria*	.	.	+	1	.
*Scorzoneroides autumnalis*	.	.	+	.	3
*Phragmites australis*	.	.	.	+	1
*Plantago lanceolata*	.	.	.	+	1
*Trifolium pratense* s.l.	.	.	.	1	1
Species of low frequency	5	6	3	2	4

**Table 5:** *Blysmus compressus* community.

Releve No.	1	2	3	4	5	6	7	8
Cover (%)	100	100	100	100	100	100	100	100
Sampled area (in sq.m)	16	16	10	16	4	4	2	4
Elevation (in m above sea level)	1367	1378	1530	1372	1383	1140	1530	1140
*Blysmus compressus* community and upper units								
*Blysmus compressus*	4	4	4	5	4	2	4	4
*Juncus articulatus* subsp. *articulatus*	2	+	1	+	1	1	1	.
*Taraxacum* Sect. *Palustria*	+	.	1	.	.	1	+	+
*Carex panicea*	2	2	.	+	.	2	.	1
*Epilobium palustre*	+	1	.	+	1	.	.	.
*Dactylorhiza incarnata* subsp. *incarnata*	+	1	.	.	1	.	.	.
*Eleocharis quinqueflora*	.	.	1	.	.	.	.	.
*Carex hostiana*	.	.	.	.	.	+	.	.
Other species								
*Ranunculus repens*	+	+	3	1	1	3	2	2
*Scorzoneroides autumnalis*	1	1	2	1	1	1	1	2
*Carex hirta*	1	1	2	.	1	+	1	2
*Deschampsia caespitosa* subsp. *caespitosa*	.	+	2	+	+	+	+	.
*Cynosurus cristatus*	.	+	.	+	+	1	1	+
*Trifolium repens*	.	.	1	.	1	.	1	2
*Ranunculus acris* s.l.	+	.	1	.	+	1	.	.
*Galium palustre* subsp. *palustre*	1	2	.	1	1	.	.	.
*Bellis perennis*	+	1	.	+	1	.	.	.
*Briza media*	2	1	.	.	.	.	.	+
*Cerastium holosteoides*	+	.	.	1	+	.	.	.
*Carex flacca* subsp. *flacca*	.	.	1	.	.	.	1	.
*Eleocharis uniglumis*	.	.	.	+	.	.	+	.
*Agrostis castellana*	.	1	+	.	.	.	.	.
*Trifolium pratense* s.l.	.	.	+	.	.	.	.	+
*Juncus inflexus* subsp. *inflexus*	.	+	.	+	.	.	.	.
*Anthoxanthum odoratum*	.	+	.	.	+	.	.	.
*Cardamine apennina*	+	.	.	+	.	.	.	.
*Glyceria notata*	+	.	.	+	.	.	.	.
*Rhinanthus minor*	+	.	.	.	.	.	.	1
*Ajuga reptans*	+	.	.	.	.	.	.	+
*Veronica beccabunga* subsp. *beccabunga*	.	.	.	+	1	.	.	.
*Epilobium parviflorum*	.	.	.	+	+	.	.	.
*Trifolium fragiferum* subsp. *fragiferum*	.	.	.	.	.	2	.	2
Species of low frequency	2	4	3	0	0	4	3	2

**Table 6:** *Menyanthetum trifoliatae* Steffen 1931.

Cover (%)	90
Sampled area (in sq.m)	4
Elevation (in m above sea level)	1140
*Menyanthetum trifoliatae* and upper units	
*Menyanthes trifoliata*	4
*Blysmus compressus*	1
*Juncus articulatus* subsp. *articulatus*	1
*Triglochin palustris*	+
*Dactylorhiza incarnata* subsp. *incarnata*	+
Other species	
*Equisetum palustre*	2
*Mentha aquatica* subsp. *aquatica*	2
*Caltha palustris*	1
*Juncus inflexus* subsp. *inflexus*	1
*Ranunculus repens*	1
*Carex hirta*	+
*Epilobium parviflorum*	+
*Galium palustre* subsp. *palustre*	+
*Hypericum tetrapterum*	+
*Lolium arundinaceum* subsp. *arundinaceum*	+
*Myosotis scorpioides* subsp. *scorpioides*	+

**Table 7:** *Carex nigra* subsp. *nigra* community.

Releve No.	1	2	3	4	5
Cover. (%)	100	100	100	100	100
Sampled area (in sq.m)	16	16	25	16	20
*Carex nigra* subsp. *nigra* community and upper units					
*Carex nigra* subsp. *nigra*	5	5	5	5	5
*Carex panicea*	.	1	+	.	.
Other species					
*Ranunculus repens*	2	1	2	1	2
*Galium palustre* subsp. *palustre*	1	+	2	1	2
*Deschampsia cespitosa* subsp. *cespitosa*	+	+	+	+	+
*Veronica scutellata*	+	1	1	+	+
*Mentha arvensis*	1	1	.	+	1
*Ranunculus pollinensis*	1	+	1	.	.
*Carex leporina*	1	.	.	+	+
*Danthonia decumbens* subsp. *decumbens*	+	.	+	.	.
*Vicia villosa*	+	.	.	+	.
*Valeriana officinalis* subsp. *officinalis*	.	.	.	+	1
*Poa trivialis*	.	.	.	+	+
*Persicaria amphibia*	+	.	.	.	.

## Data Availability

All the original data are presented in the paper. Moreover, the data will soon be stored and available at “http://www.anarchive.it/” in the sub–project CARDAV.

## Supplementary Materials

The following supporting information can be downloaded at https://www.mdpi.com/article/10.3390/plants13101282/s1, Figure S1: *Eriophorum latifolium* community; Figure S2: *Caricetum davallianae*; Figure S3: *Eleocharitetum quinqueflorae*; Figure S4: *Eleocharitetum quinqueflorae* variant with *Triglochin palustris* and *Carex oederi*; Figure S5: *Caricetum davallianae caricetosum hostianae*. Figure S6: *Menyanthetum trifoliatae*.

## Appendix A

Taxa of low frequency

Table 1—Rel. 3: Sesleria uliginosa +, Galium palustre subsp. palustre +, Holcus lanatus subsp. lanatus +, Mentha aquatica subsp. aquatica 1, Veronica anagallis–aquatica subsp. anagallis–aquatica +; rel. 5: Cirsium palustre +, Epilobium parviflorum 1, Linum catharticum s.l. +; rel. 6: Mentha arvensis +, Poa sylvicola +, Trifolium pratense s.l. +; rel. 7: Bromus racemosum subsp. racemosum 1, Carex leporina 1, Cynosurus cristatus +; rel. 8: Bellardiochloa variegata subsp. variegata +, Carex vulpina +, Plantago major +; rel 9: Juncus compressus 2; rel. 10: Eleocharis uniglumis 1, Plantago subulata +; rel. 11: Anacamptis pyramidalis +, Carex distans 1, Festuca rubra s.l. +; Lathyrus pratensis subsp. pratensis +, Phragmites australis +; rel. 12: Plantago major 2, Agrostis stolonifera 3, Eleocharis palustris 1.

Table 3—Rel. 1: Primula vulgaris subsp. vulgaris 1, Cirsium palustre +, Filipendula ulmaria +; rel. 5: Tragopogon pratensis +, Cerastium holosteoides +; rel. 6: Lolium arundinaceum subsp. arundinaceum +; rel. 8: Equisetum arvense 3, Petasites hybridus subsp. hybridus +; rel. 9: Agrostis stolonifera s.l. +; rel. 13: Anthoxanthum odoratum +; rel. 14: Danthonia decumbens subsp. decumbens +, Nardus stricta +, Juncus conglomeratus +, Carex pallescens +, Veronica scutellata +; rel. 15: Eleocharis uniglumis +; rel. 16: Trifolium montanum subsp. rupestre +; rel. 17: Serratula tinctoria subsp. tinctoria +; rel. 18: Genista tinctoria +; rel. 19: Medicago lupulina +, Caltha palustris +, Rorippa sylvestris subsp. sylvestris +; rel. 20: Cynosurus cristatus 1, Angelica sylvestris subsp. sylvestris 1, Poa trivialis 1, Mentha longifolia +, Molinia arundinacea +, Vicia villosa +.

Table 4—Rel. 1: Ranunculus acris s.l. +, Equisetum arvense 1, Molinia caerulea 1, Bellis perennis 1, Dactylorhiza maculata subsp. saccifera +; rel. 2: Holcus lanatus subsp. lanatus 1, Cirsium palustre +, Angelica sylvestris subsp. sylvestris +, Epilobium parviflorum +, Mentha aquatica subsp. aquatica 1, Valeriana officinalis subsp. officinalis +; rel. 3: Ajuga reptans +, Mentha arvensis +, Lotus corniculatus subsp. corniculatus +; rel. 4: Anacamptis laxiflora +, Scorzoneroides cichoracea 1; rel. 5: Carex elata subsp. elata 2, Filipendula vulgaris 1, Geum rivale +, Linum catharticum s.l. +.

Table 5—Rel. 1: Alchemilla cfr. glabra 1, Phleum nodosum +; rel. 2: Ranunculus apenninus +, Lathyrus pratensis subsp. pratensis +, Linum catharticum s.l. +, Potentilla reptans +; rel. 3: Eleocharis palustris subsp. palustris 2, Lotus pedunculatus 1, Mentha arvensis 1; rel. 6: Alopecurus rendlei 3, Equisetum fluviatile 1, Carex spicata subsp. spicata +, Centaurea jacea s.l. +.

## Appendix B

Sources of data.

Table 1

Rels. 1, 2, 8: 22 June 2023 Pian Piccolo of Castelluccio (Norcia, Perugia); rels. 3, 4: 30 June 2023 Campo di Rovere (Rocca di Mezzo, L’Aquila); 31 July 2022 Vomano River springs (Pizzoli, L’Aquila); rels. 6, 12: 3 July 2023 Campo di Rovere (Rocca di Mezzo, L’Aquila); rel. 7: 4 July 2023 Campo di Rovere (Rocca di Mezzo, L’Aquila); rels. 9, 10: 20 July 2023 Campo Felice (Lucoli, L’Aquila); rel. 11: 4 June 2021 Piani di Monte Lago (Sefro, Macerata).

Table 2

Rel. 1: 8 August 2023 Voltigno Valley (Carpineto della Nora, Pescara).

Table 3

Rel. 1: 20 June 2020 between Campotosto (L’Aquila) and Amatrice (Rieti); rels. 2, 3, 4, 22 June 2023: Pian Piccolo di Castelluccio (Norcia, Perugia), rell. 5, 9, 10, 11, 12, 13: rell. 6, 1, 2, 3, 4, 5 of Table 1, respectively, from Pedrotti and Cortini Pedrotti (1982) [33]; rels. 6, 8: 31 July 2022 Vomano River springs (Pizzoli, L’Aquila); rel. 7: 05 August 2023 Pian Piccolo di Castelluccio (Norcia, Perugia); rel. 14: rel. 1 of Table 8 in Pedrotti and Murrja (2020) [35]; rel. 15, 16, 17, 18: 3 July 2023 Campo di Rovere (Rocca di Mezzo, L’Aquila); rels. 19, 20: 27 June 2020: Ponticello (Montereale, L’Aquila).

Table 4

Rels. 1, 2: 31 July 2022 Vomano River springs (Pizzoli, L’Aquila); rels. 3, 4, 5: 4 June 2021 Piani di Monte Lago (Sefro, Macerata).

Table 5

Rels. 1, 2, 4, 5: 08/08/2023 Voltigno Valley (Carpineto della Nora, Pescara); rels. 3, 7: 20 July 2023 Campo Felice (Rocca di Cambio, L’Aquila); rel. 6: 27 June 2020: Ponticello (Montereale, L’Aquila); rel. 8: 20 June 2020 between Campotosto (L’Aquila) and Amatrice (Rieti).

Table 6

Rel. 1: 31 July 2022 Vomano River springs (Pizzoli, L’Aquila).

## References

[1] https://environment.ec.europa.eu/topics/nature-and-biodiversity/habitats-directive_en.

[2] https://environment.ec.europa.eu/strategy/biodiversity-strategy-2030_en.

[3] https://environment.ec.europa.eu/topics/nature-and-biodiversity/birds-directive_en.

[4] Biondi E., Burrascano S., Casavecchia S., Copiz R., Del Vico E., Galdenzi D., Gigante D., Lasen C., Spampinato G., Venanzoni R. (2012). Diagnosis and syntaxonomic interpretation of Annex I Habitats (Dir. 92/43/EEC) in Italy at the alliance level. Plant Sociol..

[5] European Commission (2013). Interpretation Manual of European Union Habitats.

[6] Bracco F., Venanzoni R. (2004). Introduzione. Quad. Habitat.

[7] Bracco F., Venanzoni R. (2004). La vegetazione delle torbiere. Quad. Habitat.

[8] Mucina L., Bultmann H., Dierßen K., Theurillat J.-P., Raus T., Carni A., Šumberová K., Willner W., Dengler J., Garcıa R.G. (2016). Vegetation of Europe: Hierarchical floristic classification system of vascular plant bryophyte lichen and algal communities. Appl. Veg Sci..

[9] Biondi E., Blasi C., Allegrezza M., Anzellotti I., Azzella M.M., Carli E., Casavecchia S., Copiz R., Del Vico E., Facioni L. (2014). Plant communities of Italy: The vegetation Prodrome. Plant Biosyst..

[10] Herbichowa M., Wołejko L., Herbich W.J. (2004). Górskie i nizinne torfowiska zasadowe o charakterze młak turzycowisk i mechowisk. Wody Słodkie i Torfowiska. Poradniki Ochrony Siedlisk i Gatunków Natura 2000.

[11] Bracco F., Stoch F., Minelli A., Venanzoni R. (2004). Aspetti di conservazione e gestione. Quad. Habitat.

[12] Pedrotti F. (2019). Flora e vegetazione della Palude di Colfiorito (Appennino Centrale, Italia). Les Cah. Braun–Blanquetia.

[13] Berisha N., Ćušterevska R., Millaku F., Matevski V. (2023). *Blysmo compressi*–*Eriophoretum latifoliae* ass. nova, a new association of the *Caricion fuscae* alliance from the Sharri Mountains. Plant Sociol..

[14] Poldini L. (1973). Die Pflanzendecke der Kalkflachmoore in Friaul (Nordostitalien). Veröff. Geobot. lnst. Rübel.

[15] Barberis G., Mariotti M. (1981). Ricerche geobotaniche sulle zone umide del Gruppo di Voltri (Appennino ligure–piemontese). Arch. Bot. Biogeogr. Ital..

[16] Marchiori S., Sburlino G. (1982). I prati umidi dell’anfitealro morenico del Tagliamento (Friuli—Italia nord–orientale). Doc. Phytosociol. N. S..

[17] Gerdol R., Tomaselli M. (1987). Mire Vegetation in the Apuanian Alps (Italy). Folia Geobot. Phytotaxon..

[18] Gerdol R., Tomaselli M. (1997). Vegetation of wetlands in the Dolomites. Diss. Bot..

[19] Ferrari C., Manzini M.L. (1987). Osservazioni sulla vegetazione macrofitica del Lago Calamone (Appennino settentrionale). Inform. Bot. Ital..

[20] Balátová–Tulácková E., Venanzoni R. (1989). Sumpf und feuchtrasengesellschaften in der verlandungszone des kalterer sees (Lago di Caldaro), der montiggler (Monticolo) seen und in der etsch (Adige) aue, Oberitalien. Folia Geobot. Phytotaxon..

[21] Balátová–Tulácková E., Venanzoni R. (1990). Beitrag zur Kenntnis der Nab–und Feuchtwiesen in der montanen Stufe der Provinz Bozen (Bolzano) Italien. Tuexenia.

[22] Sburlino G., Ghirelli L. (1994). Le cenosi a *Schoenus nigricans* L. del *Caricion davallianae* Klika 1934 nella Pianura padana orientale (Veneto–Friuli). Stud. Geobot..

[23] Gerdol R. (1994). La vegetazione degli ambienti umidi delle Alpi Carniche meridionali. Gortania–Atti Mus. Friul. Sci. Nat..

[24] Gerdol R., Tomaselli M., Bragazza L. (1994). A floristic–ecologic classification of five mire sites in the montane–subalpine belt of south Tyrol (S Alps, Italy). Phyton.

[25] Minghetti P., Pedrotti F. (2000). La vegetazione del Laghetto delle Regole di Castelfondo (Trento). Studi Trentini Sci. Nat. Acta Biol..

[26] Foggi B., Gennai M., Gervasoni D., Ferretti G., Rosi C., Viciani D., Venturi E. (2007). La carta della vegetazione del SIC Alta Valle del Sestaione (Pistoia, Toscana Nord–Occidentale). Parlatorea.

[27] Vanacore Falco I., Venanzoni R. (2009). Indagine floristico–vegetazionale nel Sito d’Importanza Comunitaria “Talweg della Val Ferret” (IT 1204032) (Courmayeur, Aosta). Rev. Valdôtaine D’histoire Nat..

[28] Gentile S. (1982). Zonation altitudinale della végétation de l’Italie méridionale et en Sicilie (Etna exclu). Ecol. Medit..

[29] Pedrotti F. (1967). Carta fitosociologica (1:3000) della vegetazione del Piani di Montelago (Camerino). Notiz. Fitosociol..

[30] Pedrotti F., Sanesi G. (1969). Resoconto sulle escursioni dell’Appennino Umbro–Marchigiano (25–27 giugno 1968). Mitt. Ostalp.–Dinar. Pflanzensoziol. Arbeitsgem.

[31] Pedrotti F., Pettorossi L. (1969). Rilevamento cartografico della vegetazione della palude di Colfiorito. Mitt. Ostalp.–Dinar. Pflanzensoziol. Arbeitsgem.

[32] Pedrotti F., Pedrotti F. (1982). La végétation des Monts de La Laga. Guide–Itinéraire. Excursion Internazionale de Phytosociologie en Italie Centrale (2–11 Juillet 1982).

[33] Pedrotti F., Cortini Pedrotti C., Pedrotti F. (1982). Les “Cariceta” du Pian Perduto de Gualdo. Guide–Itinéraire. Excursion Internazionale de Phytosociologie en Italie Centrale (2–11 Juillet 1982).

[34] Catorci A., Ballelli S., Gatti R., Vitanzi A. (2008). Studio fitosociologico delle praterie della Valle dell’Ambro (Parco Nazionale dei Monti Sibillini, Italia Centrale). Inf. Bot. Ital..

[35] Pedrotti F., Murrja E. (2020). Cartografia della vegetazione del laghetto del Pian Piccolo (Monti Sibillini, Appennino centrale) eseguita con l’aiuto del drone. Cah. Braun–Blanquetia.

[36] Biondi E., Ballelli S., Allegrezza M., Taffetani F., Frattaroli A.R., Guitian J., Zuccarello V. (1999). La vegetazione di Campo Imperatore (Gran Sasso d’Italia). Braun–Blanquetia.

[37] Pirone G. (2000). La vegetazione ripariale nei versanti nord–orientali del Gran Sasso d’Italia e dei Monti della Laga (Abruzzo, Italia). Fitosociologia.

[38] Gargano D., Passalacqua N.G., Bernardo L. (2007). Bogs and Mires in Mediterranean Areas: The Vegetation of the Marshlands of the Lacina Plain (Calabria, S. Italy). Phyton.

[39] Ciaschetti G., Pirone G., Venanzoni R. (2020). Sedge vegetation of the “Major Highlands of Abruzzo” (Central Italy): Updated knowledge after new discoveries. Plant Biosyst..

[40] Venanzoni R., Praleskouskaya S., Ciaschetti G. (2021). Contribution to the Syntaxonomy of Rare Tall Sedge Community in Central Apennine (Umbria–Italy): I. Caricetum buxbaumii. Flora Medit..

[41] Tarquini S., Isola I., Favalli M., Mazzarini F., Bisson M., Pareschi M.T., Boschi E. (2007). TINITALY/01: A new Triangular Irregular Network of Italy. Ann. Geophys..

[42] Lùdi W. (1921). Die Pflanzengesellschaften des Lauterbrunnentales und ihre Sukzession. Beitr. Geobot. Landesaufn..

[43] Zelnik I., Martinčič A., Vreš B. (2010). Vegetation of the depressions with *Eleocharis quinqueflora* in spring fens in Slovenia. Acta Biol. Slov..

[44] Jeschke L. (1963). Die Wasser– und Sumpfvegetation im Naturschurzgebiet “Ostufer der Muritz”. Limnologica.

[45] Braun W. (1968). Die Kalkflachmoore und ihre wichtigsten Kontaktgesellschaften im Bayerischen Alpenvorland. Diss. Bot..

[46] Dierssen K. (1982). Die wichtigsten Pflanzengesellschaften der Moore NW–Europas.

[47] Dítě D., Navrátilová J., Hájek M., Valachovič M., Pukajová D. (2006). Habitat variability and classification of *Utricularia* communities: Comparison of peat depressions in Slovakia and the Třeboň basin. Preslia.

[48] Hájek M., Hájková P., Chytrý M. (2011). Vegetation of fens, transitional mires and bog hollows. Vegetace České Republiky 3. Vodní a Mokřadní Vegetace.

[49] Brusa G. (2020). Le comunità vegetali degli habitat di interesse comunitario 7210 “Paludi calcaree con *Cladium mariscus* e specie del *Caricion davallianae*” e 7230 “Torbiere basse alcaline” in Lombardia. Nat. Brescia.–Ann. Mus. Civ. Sc. Nat. Brescia.

[50] e-veg—A Database Upon European Vegetations. https://www.e-veg.net.

[51] Pignatti S., Guarino R., La Rosa M. (2017). Flora d’Italia.

[52] Gerdol R., Tomaselli M. (1993). The vegetation of wetlands in the northern Apennines (Italy). Phytocoenologia.

[53] Raffaelli M., Mori Secci M., Mariotti Lippi M., Fiorini G. (1997). Indagini floristico–vegetazionali e actuopalinologiche sul Lago Baccio e sul Lago del Greppo (Appennino Tosco–Emiliano). Webbia.

[54] (2023). Portal to Flora of Italy. https://dryades.units.it/floritaly/.

[55] Turmel J.-M. (1955). Le pic de midi d’Ossau. Ecologie et végétation. Mém. Mus. Nat. Hist. Nat. Paris, N. S. Sér. B Bot..

[56] Moravec J., Rybníčková E. (1964). Die *Carex davalliana–Bestände* im Böhmerwaldvorgebirge, ihre Zusammensetzung, Ökologie und Historie. Preslia.

[57] Dietl W. (1975). Die landschaftsokologische Bedeutung der Flachmoore. Beispiel: Davallseggenrieder. Jahrb. Vereins Schutze Bergwelt.

[58] Moravec J. (1966). Zur Syntaxonomie der *Carex davalliana–Gesellschaften*. Folia Geobot. Phytotax..

[59] Görs S. (1963). Beiträge zur Kenntnis basiphiler Flachmoorgesellschaften (*Tofieldietalia* Preisg. apud Oberd.49). I. Teil. Das Davallseggen–Quellmoor (*Caricetum davallianae* W.Koch 28). Veröff. Württemb. Landesst. Naturschutz U. Landschaftspflege Baden–Württemb..

[60] Pedrotti F., Venanzoni R. (1987). La vegetazione di un bacino glacio–carsico sull’altipiano di Folgaria (Trentino). Webbia.

[61] Di Pietro R., Tondi G., Minutillo F., Bartolucci F., Tinti D., Cecchetti S., Conti F. (2008). Ulteriore contributo alla conoscenza della flora vascolare dei Monti della Laga (Appennino centrale). Webbia.

[62] Conti F., Bartolucci F. (2011). Notulae alla checklist della flora vascolare italiana 11. 1782–1793. Inf. Bot. Ital..

[63] Dutoit D. (1924). Les associations vegetales des Sous-Alpes de Vevey (Suisse). Ph.D. thesis.

[64] Issler E. (1932). Les associations végétales des Vosges et de la plaine rhénane avoisinante. 3. Les prairies: A. Les prairies non fumées du Ried ello–rhénan et le *Mesobrometum* du Haut–Rhin. Diagnoses phytosociologiques. Bull. Soc. Hist. Nat. Colmar.

[65] Hallberg H.P. (1971). Vegetation auf den Schalenablagerungen in Bohuslän, Schweden. Acta Phytogeogr. Suec..

[66] Rodwell J.S. (1992). British Plant Communities.

[67] Trinajstić I. (2002). Livade rane pahovke s gomoljastom končarom—As. *Filipendulo vulgaris–Arrhenatheretum* Hundt & Hübl (Arrhenatherion) u Hrvatskoj. Agron. Glas..

[68] Pignatti S., Menegoni P., Pietrosanti S. (2005). Valori di biondicazione delle piante vascolari della flora d’Italia. Bioindicator values of vascular plants of the Flora of Italy. Braun–Blanquetia.

[69] Montanari C., Guido M.A. (1980). La vegetazione idro–igrofila di alcune conche lacustri del versante nord di Monte Ragola (Alta Val Nure—Appennino Ligure–Piacentino). Arch. Bot. Biogeogr. Ital..

[70] Rivas–Martínez S., Fernández–González F., Loidi J., Lousã M., Penas A. (2001). Syntaxonomical checklist of vascular plant communities of Spain and Portugal to association level. Itinera Geobot..

[71] Hájek M., Hájková P., Apostolova I. (2008). New plant associations from Bulgarian mires. Phytol. Balc..

[72] Borhidi A., Kevey B., Lendvai G. (2012). Plant communities of Hungary.

[73] Andreis C., Zavagno F. (1996). La vegetazione del Lago di Ganna, con particolare riferimento ai rapporti spaziali tra le cenosi dei *Molinietalia* e degli *Scheuchzerietalia palustris*. Nat. Valtellin..

[74] Gerdol R. (1987). Geobotanical investigations in the small lakes of Lombardy. Atti Ist. Bot. Lab. Critt. Univ. Pavia.

[75] Borhidi A. (2003). Magyarország Növénytársulásai.

[76] Dítě D., Hájek M., Hájková P. (2007). Formal definitions of Slovakian mire plant associations and their application in regional research. Biologia.

[77] Redžić S., Barudanović S., Trakić S., Kulijer D. (2011). Vascular plant biodiversityrichness and endemo relictness of the karst mountains Prenj–Čvrsnica–Čabulja inBosnia and Herzegovina (W. Balkan). Acta Carsologica.

[78] Theurillat J.-P., Willner W., Fernández-González F., Bültmann H., Čarni A., Gigante D., Mucina L., Weber H. (2021). International Code of Phytosociological Nomenclature. 4th edition. Appl. Veg. Sci..

[79] Mariotti M.G. (2008). Atlante Degli Habitat Natura 2000 in Liguria.

[80] Koch W. (1928). Die höhere Vegetation der subalpinen Seen und Moorgebiete des Val Piora. Z. Hydrol..

[81] Quézel P. (1964). Végétation des hautes montagnes de la Grèce méridionale. Vegetatio.

[82] Coldea G. (1977). Untersuchung der basiphilen Flachmoorgesellschaften aus Rumänien (*Tofieldietalia* Prsg. apud Oberd. 1949). Phytocoenologia.

[83] Felbaba–Klushina L.M., Konishchuk V.V. Scheuchzerio palustris–Caricetea fuscae TX. 1937. In Prodrome of the Vegetation of Ukraine; Dubyna, D.V., Dziuba, T.P., Iemelianova, S.M., Bahrikova, N.O., Borysova, O.V., Borsukevych, L.M., Vynokurov, D.S., Hapon, S.V., Hapon, Y.V., Davydov, D.A., et al., Eds.; Naukova Dumka, Kyiv, Ukraine, 2019, pp. 184–197.

[84] Eggler J. (1933). Die Pflanzengesellschaften der Umgebung von Graz. Repert. Spec. Nov. Regn. Veget. Beih..

[85] Müller W. (2021). Carici–Blysmetum compressi (Eggler 33)—Eine für Deutschland Bisher Unbekannte Pflanzengesellschaft. https://www.academia.edu/74604168/Carici_Blysmetum_compressi_Eggler_33_eine_f%C3%BCr_Deutschland_bisher_unbekannte_Pflanzengesellschaft.

[86] Redžić S. (2007). Syntaxonomic diversity as an indicator of ecological diversity—Case study Vranica Mts in the Central Bosnia. Biologia.

[87] Papp B., Alegro A., Šegota V., Šapić I., Vukelić J. (2013). Contributions to the bryophyte flora of Croatia I. Gorski kotar Region (W Croatia). Stud. Bot. Hung..

[88] Šilc U., Čarni A. (2012). Conspectus of vegetation syntaxa in Slovenia. Hacquetia.

[89] Hájek M., Jiménez–Alfaro B., Hájek O., Brancaleoni L., Cantonati M., Carbognani M., Dedic A., Dítě D., Gerdol R., Hájková P. (2021). A European map of groundwater pH and calcium. Earth Syst. Sci. Data.

[90] Pignatti S., Guarino R., La Rosa M. (2018). Flora d’Italia.

[91] Šumberová K., Hájková P., Chytrý M., Hroudová Z., Sádlo J., Hájek M., Hrivnák R., Navrátilová J., Hanáková P., Ekrt L., Chytrý M. (2011). Vegetace rákosin a vysokých ostřic (*Phragmito–Magno–Caricetea*). Vegetace České Republiky 3. Vodní a Mokřadní Vegetace.

[92] Thébaud G., Roux C., Delcoigne A., Petel G. (2012). Contribution à une révision des basmarais acides d’Europe tempérée occidentale. Phytocoenologia.

[93] Vlieger J. (1937). Aperçu sur les unités phytosociologiques supérieures des Pays–Bas. Ned. Kruidkd. Arch. Ser. 3.

[94] Soó R. (1954). Die Torfmoore Ungarns in dem Pflanzensoziologischen System. Vegetatio.

[95] Valachovič M. (2001). Rastlinné Spoločenstvá Slovenska. 3. Vegetácia Mokradí.

[96] Braun-Blanquet J. (1964). Pflanzensoziologie. Grundzüge der Vegetationkunde.

[97] Van der Maarel E. (1979). Transformation of cover–abundance values in phytosociology and its effects on community similarity. Vegetatio.

[98] Burba N., Feoli E., Malaroda M., Neves R., Baretta J.W., Mateus M. (2008). MATEDIT: A software tool to integrate information in decision making processes. Perspectives on Integrated Coastal Zone Management in South America.

[99] Podani J. (2001). Syn–Tax 2000. Computer Program for Data Analysis in Ecology and Systematics.

[100] Paganelli A., Pedrotti F. (1982). Histoire paleobotanique. Guide–Itinéraire. Excursion Internationale de Phytosociologie en Italie centrale (2–11 Juillet 1982).

[101] Venanzoni R., Properzi A., Bricchi E., Landucci F., Gigante D., Pedrotti F. (2018). The *Magnocaricetalia* Pignatti 1953 (*Phragmito*–*Magnocaricetea* Klika in Klika et Novák 1941) plant communities of Italy. Climate Gradients and Biodiversity in Mountains of Italy.

