# Supplementary material for: Relicts of Threatened Biodiversity: Similarities and Differences among the 7230 EU Habitat Plant Communities on Montane Plateaus of Central Apennines, Italy
Source: Plants (Basel). 2024 May 7;13(10):1282. doi: 10.3390/plants13101282 (PMC11124865; doi:10.3390/plants13101282)
Supplement: Supplementary file 1 [file plants-13-01282-s001.zip › plants-2950002-supplementary.pdf]

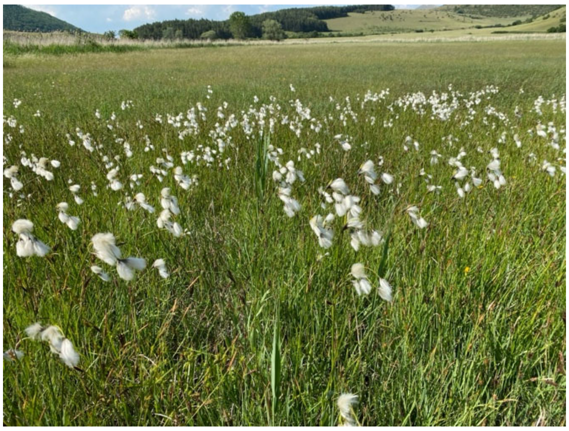

Figure S1: *Eriophorum latifolium* community.

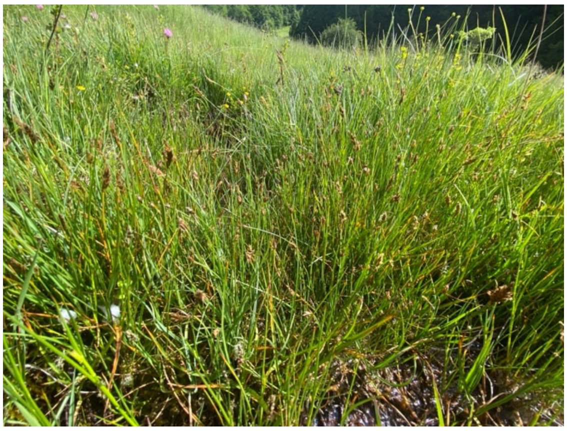

Figure S2: *Caricetum davallianae.*

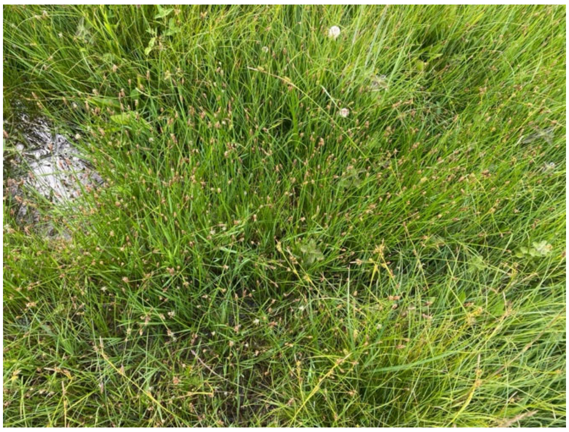

Figure S3: *Eleocharitetum quinqueflorae.*

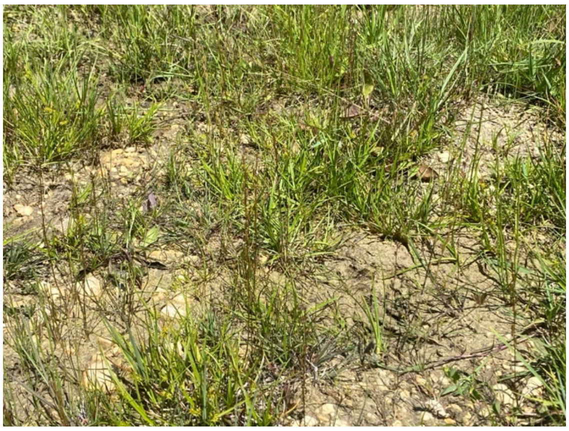

Figure S4: *Eleocharitetum quinqueflorae* variant with *Triglochin palustris* and *Carex oederi.*

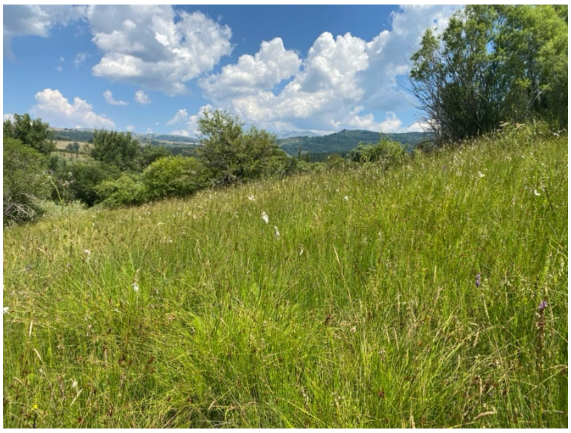

Figure S5: *Caricetum davallianae caricetosum hostianae*.

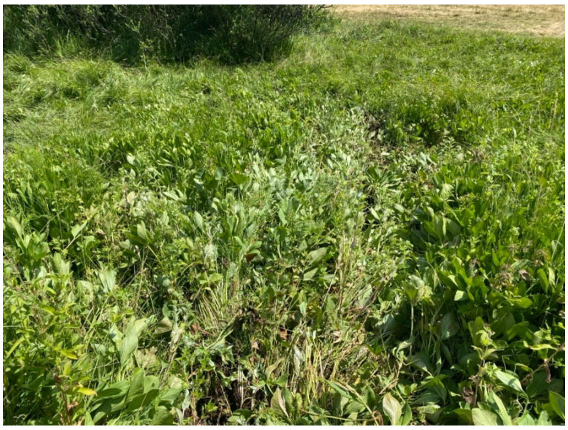

Figure S6: *Menyanthetum trifoliatae.*
